# Pentatricopeptide Protein PTCD2 Regulates COIII Translation in Mitochondria of the HeLa Cell Line

**DOI:** 10.3390/ijms232214241

**Published:** 2022-11-17

**Authors:** Maria V. Baleva, Ivan Chicherin, Uliana Piunova, Viktor Zgoda, Maxim V. Patrushev, Sergey Levitskii, Piotr Kamenski

**Affiliations:** 1 Faculty of Biology, Lomonosov Moscow State University, 1/12 Leninskie Gory, 119234 Moscow, Russia; 2 Institute of Biomedical Chemistry, Russian Academy of Sciences, 10/8 Pogodinskaya Str., 119121 Moscow, Russia; 3 National Research Centre “Kurchatov Institute”, 1 Akademik Kurchatov Square, 123182 Moscow, Russia

**Keywords:** mitochondria, translation, translation regulation

## Abstract

Protein biosynthesis in mitochondria is tightly coupled with assembly of inner membrane complexes and therefore must be coordinated with cytosolic translation of the mRNAs corresponding to the subunits which are encoded in the nucleus. Molecular mechanisms underlying the regulation of mitochondrial translation remain unclear despite recent advances in structural biology. Until now, only one translational regulator of protein biosynthesis in mammalian mitochondria is known—protein TACO1, which regulates translation of COI mRNA. Here we describe the function of pentatricopeptide-containing protein PTCD2 as a translational regulator of another mitochondrially encoded subunit of cytochrome c oxidase—COIII in the HeLa cell line. Deletion of the PTCD2 gene leads to significant decrease in COIII translation efficiency and impairment in CIV activity. Additionally, we show that PTCD2 protein is partially co-sedimentates with associated mitochondrial ribosome and associates with mitochondrial ribosome proteins in pull-down assays. These data allow concluding that PTCD2 is a specific translational regulator of COIII which attracts the mRNA to the mitochondrial ribosome.

## 1. Introduction

In the course of billions of evolution years, the mitochondria dramatically reduced the genome of their ancestors: almost all protomitochondrial genes were transferred to the nucleus or lost [1]. Nowadays, most of the genes encoding mitochondria-specific proteins are localized and transcribed in the nucleus, their mRNAs are translated in the cytosol, and the proteins are imported into mitochondria via specific protein transport machinery [2]. Nevertheless, most of modern mitochondria retained significantly reduced genomes containing limited set of genes along with the apparatus of genome maintenance and gene expression. In addition to the genes of ribosomal and transfer RNAs, mitochondrial genomes contain a set of protein-coding genes. The human mitochondrial genome contains 13 protein-coding genes, all of which encode subunits of electron transport chains [3].

Mitochondrial protein synthesis was thought to be similar to bacterial translation for decades. However, recent advances in cryo-electron microscopy and mitochondrial molecular biology revealed profound differences between the mitochondrial and bacterial protein synthesis machineries observed in the structures of ribosomes, mechanisms of translation initiation, and other aspects [3,4,5,6,7]. One of the peculiarities of mitochondrial translation worth noting is its membrane tethering, which facilitates the biosynthesis and insertion of OXPHOS subunits. As it was mentioned above, all proteins encoded in human mitochondrial DNA (mtDNA) are the subunits of electron transport chain complexes; however they altogether present a minor part of the complete set, whereas the major part is synthesized in cytosol. Therefore, cytosolic and mitochondrial translation processes must be tightly coordinated for the correct assembly of OXPHOS complexes. Such coordination is well described in baker’s yeast mitochondria, where it is modulated by a group of specific regulatory proteins—translational activators [4]. The efficiency of translation initiation of every mitochondrial mRNA of *S. cerevisiae* is governed by binding of specific translational activator to prolonged 5′-UTR and/or mitochondrial ribosome [4]. Human mitochondrial mRNAs are leaderless and do not have any significant 5′-UTRs [5]. At first glance, this fact makes translational activators unnecessary; however, the existence of analogous regulatory system cannot be excluded.

Pentatricopeptide-containing proteins (PPR proteins) are good candidates for the role of the regulators of mRNA translation in human mitochondria. These proteins belong to the α-solenoid RNA-binding proteins involved in almost all steps of the life cycle of mRNAs [6]. All PPR proteins contain varying numbers of tandemly repeated sequence motifs of 35 amino acids (the PPR motifs) and are abundant in plant organelles but also found in all eukaryotic lineages [7]. Proteins containing PPR motifs are known to have roles in transcription, RNA processing, splicing, stability, editing, and translation. Many yeast mitochondrial translational activators are PPR proteins [4]. The human genome contains several genes encoding mitochondrial PPR proteins, e.g., *POLMT*, *LRPPRC*, *PTCD1*, *PTCD2*, *PTCD3*, *MRPS27*, etc. [8].

The focus of our research is PTCD2, a mysterious protein whose function was unknown despite the available data. Its mitochondrial localization and PPR family membership suggested its possible biological role in regulation of mitochondrial gene expression. A PTCD2 knock-out mouse model was studied previously. It demonstrated the reduction in activity of complex III in the heart, an increase in activity of complex IV in heart, muscle and liver along with defects in apocytochrome b mRNA maturation [9]. However, the published model was not a true knock-out because truncated protein was still synthesized, and only exons 9 and 10 were missing. We made disruption of PTCD2 gene in human HeLa cells by introducing a frame-shift mutation close to the start codon and analyzed their mitochondrial translation, oxygen consumption, OXPHOS complex activity, and supercomplex formation. We show that PTCD2 deletion selectively decreased translation of COIII mRNA and the activity of the respiratory chain complex IV (CIV). As a consequence, it affects respiration and supercomplex formation. In addition, we obtained the data from sedimentation analysis and pull-down proteomics which allow us to suggest the interaction of PTCD2 with mitochondrial ribosomes.

## 2. Results

### 2.1. Deletion in Nuclear PTCD2 Gene Leads to Selective Decrease in COIII mRNA Translation in Human Mitochondria

**Figure 1:**
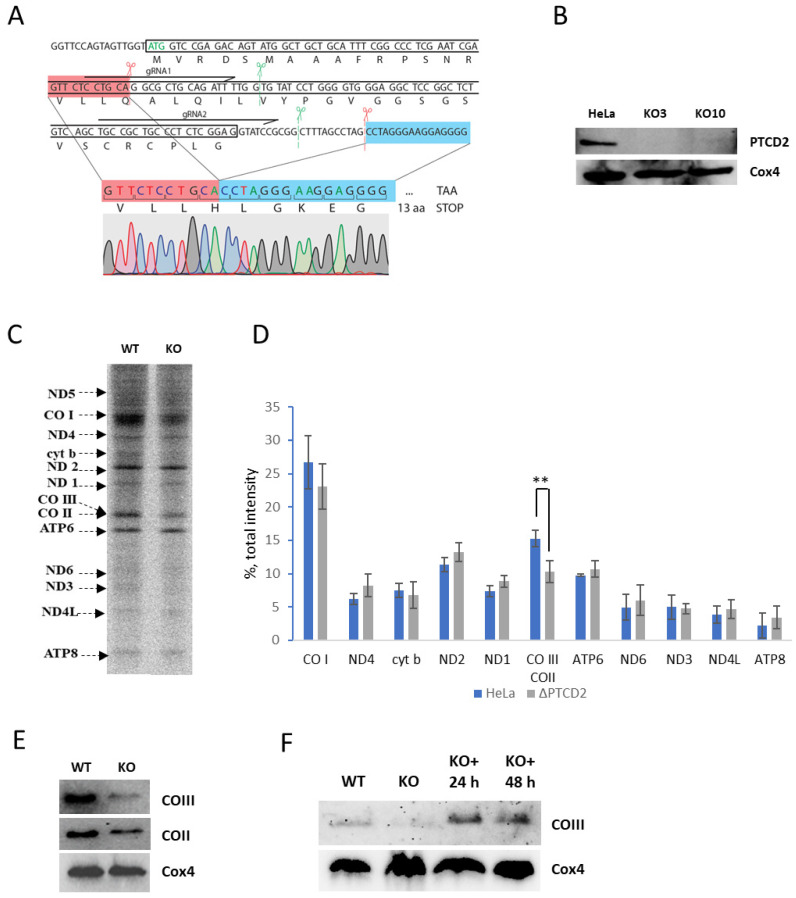
Functional deletion of *PTCD2* gene affects translation and steady-state level of COIII protein in human mitochondria. (**A**) *PTCD2* gene disruption with CRISPR/Cas9 technology. The sequence of exon 1 is shown in a black box. Start codon is green. SgRNA sequences are shown as black horizontal arrows indicating the direction from 5′ to 3′. Green scissors with vertical dashed lines show the intended positions of Cas9 cleavage. Red scissors with vertical dashed lines show the real positions of Cas9 cleavage. The DNA ends joint together after Cas9 cleavage and DNA repair are shown in red and blue boxes, and the region between them was deleted, which is proved by sequencing chromatogram. (**B**) Western blot analysis of wild-type HeLa and *PTCD2* knock-out cell lysates with antibodies against PTCD2 and Cox4. (**C**) Mitochondrial translation products of HeLa (WT) and knock-out cells (KO). Cytosolic translation was blocked by cycloheximide and cells were exposed to ^35^S-methionine as described in Materials and Methods. Newly synthesized proteins are designated at left, representative radioautograph shown (*n* = 4). (**D**) Quantification of mitochondrial translation products in wild-type HeLa and *PTCD2* knock-out cells. Mean values ± SD are shown, *n* = 4, **—*p*-value < 0.01 by Student’s test. (**E**) Western-blot analyses of HeLa and PTCD2 knock-out cells lysates with antibodies to COIII, COII and Cox4. (**F**) Phenotype restoration. Western blot analyses of HeLa (WT), knock-out (KO), knock-out with insertion of *PTCD2* cDNA induced with doxycycline for 24 h (KO+ 24 h), and 48 h cells lysates with antibodies against COIII and PTCD2.

We produced a HeLa cell line with a functional deletion in the PTCD2 gene in order to find out whether the corresponding protein is involved in the translational regulation in human mitochondria. We utilized CRISPR/Cas9 genome editing technology to achieve this aim. We designed two spacer sequences of sgRNAs which were supposed to target the Cas9 nuclease to exon 1 of PTCD2, inserted them in a plasmid vector containing the scaffold sequence of sgRNA, and then transfected HeLa cells with a mixture of these genetic constructs and a plasmid bearing the genes for the Cas9 nuclease and GFP protein. GFP-positive cells were selected and genotyped for the desired deletions by PCR and Sanger sequencing. As a result, we obtained two cell lines (clones 3 and 10) with homozygous deletion in the first exon of PTCD2 gene causing a frame-shift mutation (Figure 1A). The DNA rearrangement which we detected in edited cell lines was not the same as we designed; however, it effectively caused the PTCD2 gene disruption due to the following considerations. First, the original start codon was intentionally preserved to avoid alternative translation initiation. Second, the deleted region was not a multiple of three, which caused a frame-shift followed by a stop codon. The absence of PTCD2 in mitochondria of mutant cell lines was confirmed by immunoblotting with PTCD2-specific antibodies (Figure 1B).

Next, we analyzed de novo mitochondrial protein synthesis in cells with deletion in *PTCD2* gene. We blocked cytosolic translation by cycloheximide and labeled mitochondrial translation products by ^35^S-methionin. Cell lysates were subjected to gel electrophoresis and radioautography and the intensities of the bands corresponding to each of the mitochondrially encoded proteins were determined (with the exception of ND5, which was poorly resolved) (Figure 1C,D). Our data based on the results of four independent experiments suggest that the absence of the PTCD2 protein in mitochondria caused a significant decrease by ~40% in the intensity of the band corresponding to the COII and COIII proteins, which poorly separate one from another by SDS-PAGE. We performed a Western blot analysis with the antibodies to both of them to find out which one was affected. It turned out that steady-state levels of both proteins were reduced, but the decrease in COIII was much more pronounced (Figure 1E). The decrease in steady-state level of COII may probably be explained by indirect consequences of *PTCD2* gene disruption, such as distortions in complex IV assembly and consequent decrease in protein life-time.

We reintroduced the *PTCD2* gene in the knock-out cell line to confirm that the effects we observed were specifically caused by the deletion and not by off-target mutations. For this reason, we integrated the cDNA copy of *PTCD2* open reading frame tagged with c-Myc epitope under the control of the tetracyclin-inducible promoter using the Sleeping Beauty transposon. After incubation of the “rescued” cell line with doxycycline for 24 h, the amount of COIII in mitochondria became comparable to that in the mitochondria of the wild-type cell line (Figure 1E). Thus, the expression of *PTCD2* gene in cells with a deletion led to the restoration of the phenotype, which indicates the specificity of the observed decrease in COIII translation.

### 2.2. The Absence of PTCD2 Affects Mitochondrial Respiration, Complex IV Activity, and Supercomplex Formation

**Figure 2:**
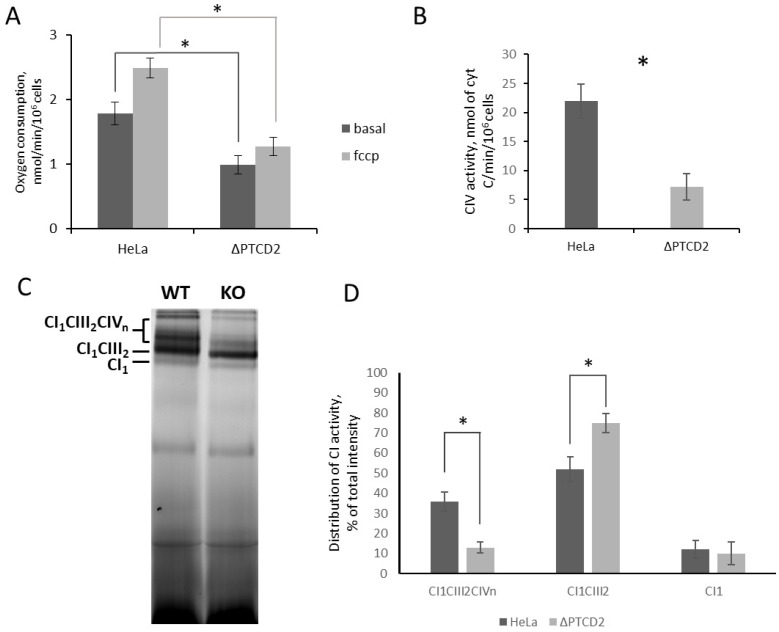
The absence of PTCD2 decrease mitochondrial fitness. (**A**) Oxygen consumption rates of wild-type HeLa and *PTCD2* gene knock-out cells in the absence (basal) and the presence (fccp) of uncoupler reagent. Mean values ± SD are presented (*n* = 6). (**B**) Activity of complex IV in HeLa and *PTCD2* gene knock-out cells. Mean values ± SD are presented (*n* = 4). (**C**) Blue-Native (4–10%) electrophoresis of mitochondria solubilized with digitonin. Gel was stained for complex I activity as described in Materials and Methods. Stoichometrical composition of resolved supercomplexes shown, WT—wild-type HeLa mitochondria, KO—*PTCD2* gene knock-out mitochondria. (**D**) distribution of CI activities between supercomplexes (*n* = 4). *—*p*-value < 0.05 by Student’s test.

Next we decided to figure out how the absence of PTCD2 affects the functionality of mitochondria. For this reason, we determined the oxygen consumption rate (OCR) of the cells with a deletion in the *PTCD2* gene and compared it with wild-type HeLa cells. The basal and maximal (in the presence of the uncoupler reagent carbonyl cyanide 4-(trifluoromethoxy)phenylhydrazone, FCCP) oxygen uptake rates were assessed. According to our data, a deletion in the *PTCD2* gene led to a decrease in basal respiration by ~45% and maximal respiration by ~50% in comparison to wild-type cells (Figure 2A). It should be noted that in knock-out cells, the basal and maximal OCRs were not significantly different, which indicates the maximal workload of complex IV in the absence of the uncoupling reagent.

Next we analyzed the activity of complex IV in *PTCD2* gene knock-out cells and compared it with wild-type HeLa cells. The KCN-dependent cytochrome c oxidase activity in cells with the deletion was reduced by approximately three-fold compared to wild-type cells (Figure 2B).

Complexes of the OXPHOS chain can organize into higher-order structures—supercomplexes—which are suggested to increase the efficiency of the OXPHOS activity by optimal substrate tunneling between the enzymes due to their spatial proximity [10]. The supercomplexes have a stoichiometry of CI_1_CIII_2_CIV_n_, and their distribution can be analyzed by Blue-Native gel electrophoresis. We performed such analysis for knock-out mitochondria versus wild-type cell mitochondria, followed by Nitro Blue tetrazolium (NBT) gel staining for complex I activity. According to our data, the number of high-order supercomplexes is significantly reduced in the mitochondria of cells with *PTCD2* gene deletion, and the activity of complex I is more concentrated in supercomplexes with CI_1_CIII_2_ stoichiometry (Figure 2C,D).

### 2.3. PTCD2 Co-Sedimentates with Associated Mitochondrial Ribosome

**Figure 3:**
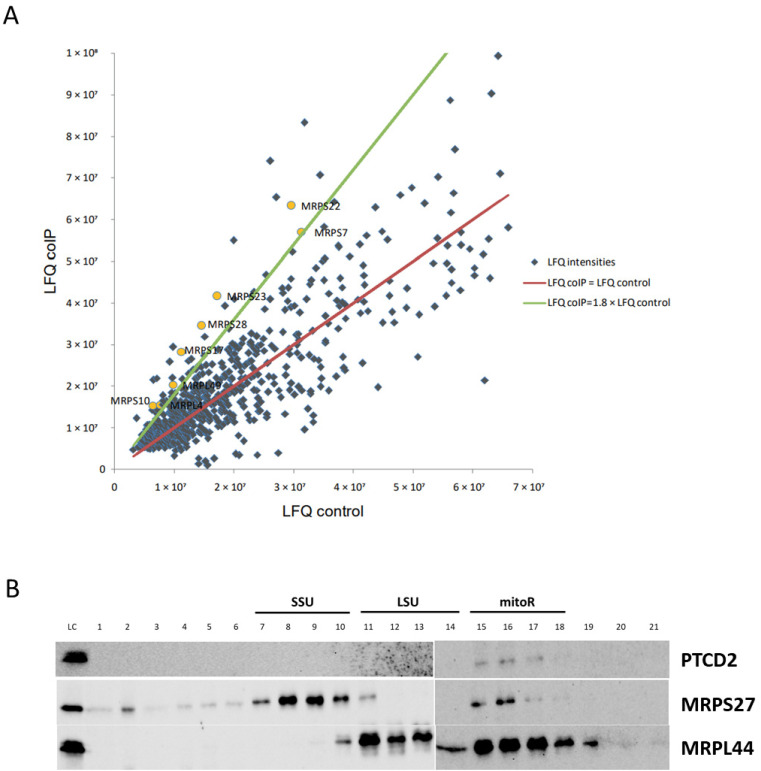
PTCD2 interacts with associated mitochondrial ribosomes. (**A**) Proteomic data analysis. The diagram represents the protein species identified in the samples of coIP and negative control. Label free quantification (LFQ) intensities for all proteins were calculated with Max Quant software. LFQs of the proteins in coIP samples are placed along the vertical axis; LFQs of the same proteins in negative control samples are placed along the horizontal axis. Blue dots show each individual protein. Yellow dots indicate mitochondrial ribosomal proteins. Red line shows the position of the points where proteins are equally represented in coIP and negative control. Green line is a threshold line showing the positions of the points where proteins are 1.8 times enriched in coIP relative to control. (**B**) Western blot analysis of fractionated mitochondrial ribosomes. Clarified HeLa cell lysate was applied to 10–30% linear sucrose gradient and ultracentrifuged. The gradient was fractionated from top to bottom in 21 fractions and proteins from each fraction were precipitated and applied to Western blot with the indicated antibodies. LC—loading control (0.1% from total lysate volume), SSU—mitoribosome small subunit, LSU—mitoribosome large subunit, mitoR—associated mitoribosome.

It was assumed previously that PTCD2 is involved in the maturation of the mitochondrial pre-mRNA of apocytochrome b and ND5 [9]. However, our data directly indicate the effect of PTCD2 on the translation of the mitochondrially encoded COIII protein. We decided to find protein interactors of PTCD2 using proteomic analysis to understand how it regulates mitochondrial translation. We produced and purified recombinant 6xHis-tagged PTCD2, incubated it with mitochondrial lysate, and co-immunoprecipitated with anti-His tag antibodies. Comparative proteomic analysis revealed a significant (>1.8-fold) enrichment in proteins of the small and large subunits of the mitochondrial ribosome among proteins co-immunoprecipitated with PTCD2 (Figure 3A).

These data suggest that PTCD2 interacts with mitochondrial ribosome. To test this hypothesis, we fractionated mitochondrial lysates by ultracentrifugation in a sucrose gradient and performed Western blot analysis of the resulting fractions with antibodies to PTCD2, MRPS27 (small subunit protein), and MRPL44 (large subunit protein) (Figure 3B). PTCD2 was detected in fractions corresponding to associated mitochondrial ribosome but not in SSU or LSU fractions.

## 3. Discussion

Regulation of human mitochondrial translation is far from being completely explained despite recent advances in structural biology providing insights in different stages of the life cycle of mitochondrial ribosomes. The molecular mechanisms governing the expression of each individual human mitochondrial mRNA and orchestrating its cross-talk with cytosolic translation are poorly studied. Such regulation in baker’s yeast is carried out via translational activators—special proteins which bind predominantly 5′-UTRs of mRNAs and increase its affinity to mitochondrial ribosome. Due to the absence of prolonged 5′-UTRs in human mitochondrial mRNAs, the existence of such system in human mitochondria has been questioned. Nevertheless, it was shown in 2009 that protein TACO1 selectively regulates the mitochondrial translation of COI mRNA [11]. The mechanism of such regulation remains unclear but provides a proof of principle for the existence of translational activators for other mRNAs. The good candidates for being translational activators are pentatricopeptide-containing proteins (PPR proteins). In this work we probed PTCD2 PPR protein for its ability to affect mitochondrial translation in human HeLa cell line mitochondria.

We found that functional deletion in the *PTCD2* gene significantly decreased translation efficiency of COIII mRNA and led to reduced steady-state level of this mitochondrially encoded protein. The decrease in intensity observed in the band corresponding to COII/COIII in radio autography could not be attributed to any of these two proteins, but steady-state level of COIII assessed by Western blotting decreased much more significantly than COII. It suggests that a decrease in COII stationary level was caused by partial loss of COIII and incorrect assembly of cytochrome c oxidase.

The decrease in the amount of COIII, which is one of CIV subunits, should affect CIV activity and overall mitochondrial fitness. We found that the absence of PTCD2 in mitochondria of HeLa cells leads to significantly lower oxygen consumption rates in the presence and absence of uncoupler reagent compared to wild-type cells. Moreover, the OCRs of knock-out cells in both conditions were equal whereas in wild-type cells these parameters usually differ double to triple. This could be explained by the fact that normally CIV has an excess capacity necessary for fast adaptation of OXPHOS to changes in the environment [12]. The amount of CIV is reduced in *PTCD2* gene knock-out cells, and therefore all CIV capacity is in use at coupled conditions.

Almost all translational activators in baker’s yeast mitochondria interact with mitoribosome and/or mRNAs. Using coIP and proteomic approaches, we found that PTCD2 interacts with a portion of SSU and LSU proteins. This fact allows suggesting that PTCD2 is associated with mitochondrial translation apparatus. Moreover, PTCD2 was found co-sedimentated with associated mitoribosome but not with SSU or LSU alone. These observations suggest that PTCD2 could interact with a part of translating mitoribosomes. However, we cannot conclude the direct interaction between PTCD2 and mitochondrial ribosome as they both could interact with mRNA. We tried to detect any interactions of recombinant PTCD2 with mRNAs (COII, COIII, COI, CytB, and ND5) by several EMSA approaches, but in our experimental conditions such interactions were not found (Probably, PTCD2–mRNA interaction requires some additional factors, e.g., mitochondrial ribosome, CIV assembly factors, or other proteins. On the other hand, the molecular mechanism of translational regulation by PTCD2 does not necessarily involve its direct interaction with mRNA. It is possible that interaction of PTCD2 with associated mitoribosome is required for efficient elongation rather than COIII mRNA recognition. We also checked the assumption that decreased levels of COIII could be the result of PTCD2 being an assembly factor of CIV. In Blue-Native Western blots PTCD2 was not detected in association with any complexes.

Another explanation for PTCD2 role in COIII translation may be based on the presence of MRPS27 domain in its structure. It can be assumed that PTCD2 can weakly compete with MRPS27 for binding to mitochondrial ribosome, and mitoribosomes which contain PTCD2 translate COIII mRNA more efficiently. Nevertheless, the precise molecular mechanism of PTCD2 action should be studied during further research.

The latest published data based on mitochondrial ribosome profiling have shown that translation initiation of COIII in human mitochondria can be carried out from completely processed leaderless and unprocessed mRNA with prolonged 5′-UTR [13]. The frequency of COIII mRNA translation initiation at processed or unprocessed transcripts varies across cell types. It could be assumed that PTCD2 is involved in initiation on one of these templates, which explains the reduction but not the complete arrest of COIII mRNA translation in our experiments. Moreover, the mode of PTCD2 action could be tissue-specific.

In summary, we have shown that deletion of PTCD2 leads to decrease in COIII translation in mitochondria, impairs the activity of CIV, OCR, and supercomplex composition in human HeLa cell mitochondria. Moreover, PTCD2 could be detected within the fraction of associated mitochondrial ribosomes and could bind mitoribosomal proteins in pull-down assays. Taken together, these facts allow us to suppose that PTCD2 can selectively regulate COIII translation in human HeLa cell mitochondria.

## 4. Materials and Methods

### 4.1. Strains, Media, Cell Lines

*E. coli* strains Top-10 One Shot (Thermo Scientific, Waltham, MA, USA) and BL21(DE3)/pLysS (Promega, Madison, WI, USA) were used for standard cloning and protein expression procedures. Human cell lines, HeLa (CCL-2 ATCC) and its derivatives, were cultured in DMEM (Corning, New York, NY, USA) with 4.5 g/L glucose, Corning @glutargo, sodium pyruvate, and 10% (*v*/*v*) FBS (Sigma, St. Louis, MO, USA), under a 5% CO_2_ humidified atmosphere at 37 °C. To prevent contamination DMEM was supplied with 1000 units/mL of penicillin, 100 µg/mL of streptomycin, and 2.5 µg/mL of Amphotericin B (all Sigma, St. Louis, MO, USA).

### 4.2. Generation of Knock-Out Cell Lines

The generation of knock-out cell lines with the CRISPR/Cas9 technology was performed as previously described in [14]. sgRNA design was performed using CRISPOR online platform (http://crispor.tefor.net (accessed on 2 October 2021)) [15]. The selected sgRNA spacer cDNA sequences (5′-CACCGCAAAATCTGCAGCGCCTGCG-3′ and 5′-AAACGCAGGCGCTGCAGATTTTGCG-3′ for sgRNA1 and 5′-CACCGGCCCTCTCGGAGGTATCCG-3′ and 5′-AAACCGGATACCTCCGAGAGGGCC-3′ for sgRNA2) were synthesized (Evrogen, Moscow, Russia) and inserted between BbsI sites in the pU6-gRNA vector containing sgRNA scaffold (kindly provided by Dr. Boris Skryabin, University of Munster). HeLa cells were co-transfected with sgRNAs-pU6 vectors together with an Cas9/EGFP-containing plasmid [16] using Lipofectamine 3000 reagent (Thermofisher Scientific, Waltham, MA, USA). Cells were sorted using FACS Aria SORP (Beckton Dickinson Biosciences, East Rutherford, NJ, USA). The gene editing was confirmed by PCR with specific primers (5′-GGACGAATCCCTTTTGTCGC-3′ and 5′-ATGCTAGCTGGGATCTAACAC-3′) followed by Sanger sequencing of resulting products.

### 4.3. Generation of Stable Cell Lines

Cell line expressing C-terminal c-Myc-tagged *PTCD2* under the control of a tetracycline-inducible promoter were generated in *PTCD2* gene knock-out HeLa cell line. Cells were co-transfected with the pSBtet-Neo vector (pSBtet-Neo was a gift from Eric Kowarz (Addgene plasmid #60509; http://n2t.net/addgene:60509 (accessed on 1 November 2022); RRID:Addgene_60509)) containing the sequence of *PTCD2* gene with C-terminal c-Myc-tag, and the pCMV(CAT)T7-SB100 vector (pCMV(CAT)T7-SB100 was a gift from Zsuzsanna Izsvak (Addgene plasmid #34879; http://n2t.net/addgene:34879 (accessed on 1 November 2022); RRID:Addgene_34879)) using Lipofectamin 3000 reagent (Thermofisher Scientific, Waltham, MA, USA). Selection of transfected cells was performed with G418 (1 mg/mL) for 14 days following cultivation in the media supplemented with 100 ng/mL of G418. The insertion of *PTCD2*-c-myc was confirmed by PCR followed by detection of c-myc tagged protein after doxycycline induction by Western blotting.

### 4.4. Mitochondrial Translation Assay

Mitochondrial translation products were labeled as described in [16]. Briefly, cells were pre-starved with methionine-free DMEM supplemented with 10% dialyzed FBS. Cytosolic translation was inactivated with 0.2 mg/mL of cycloheximide for 5 min. Then 0.5 mCi/mL [^35^S] methionine was added. After incubation at 37 °C for 45 min the medium was replaced with fully supplemented DMEM containing cold methionine and the cells were harvested. Samples were further analyzed by 18% SDS-PAGE and autoradiography. Signals were recorded on Storage Phosphor screens using a STORM 865 scanner (GE Healthcare, Marlborough, MA, USA). ImageJ software (NIH, Bethesda, MD, USA) was used for quantification.

### 4.5. Western Blot

Western blot analyses were performed conventionally using following antibodies: anti-PTCD2 (NBP2-26176, Novusbio, Centennial, CO, USA), anti-MRPS27 (17280-1-AP, Proteintech, Manchester, UK), anti-MRPL44 (16394-1-AP, Proteintech, Manchester, UK), anti-Cox4 (11242-1-AP, Proteintech, Manchester, UK), anti-COII (55070-1-AP, Proteintech, Manchester, UK), anti-COIII (55082-1-AP, Proteintech, Manchester, UK), anti-goat IgG HRP conjugated (HAF017, Novusbio, Centennial, CO, USA), ECL Anti-rabbit IgG HRP linked (NA934V, GE Healthcare, Marlborough, MA, USA), according to manufacturers’ protocols. Blots were stained with SuperSignal West Pico ECL reagents (ThermoFisher Scientific, Waltham, MA, USA) and visualized with ChemiDoc imaging system (BioRad, Hercules, CA, USA).

### 4.6. Isolation of Mitochondria

The isolation of human mitochondria was performed according to [17]. Cells were harvested, washed with PBS, and resuspended and swelled for 20 min in RSB Hypo buffer (10 mM NaCl, 10 mM MgCl_2_, 1.5 mM Tris-HCl, pH 7.5). Cells were homogenized by tight pestle in a Dounce homogenizer. After addition of 2.5xMS Buffer (525 mM mannitol, 175 mM sucrose, 2.5 mM EDTA, 12.5 mM Tris-HCl, pH 7.5), cell debris was pelleted at 1300× *g* for 5 min at 4 °C. The mitochondria were pelleted by centrifugation at 18,000× *g* for 15 min at 4 °C. Mitochondria quantity was estimated by measurement of total mitochondria protein concentration by Bradford assay. Mitochondria pellets were stored at −80 °C until use.

### 4.7. Blue-Native Electrophoresis

BN-PAGE was performed as described in [18]. Briefly, 100 μg of isolated mitochondria were resuspended in 10 μL of solubilization buffer A (50 mM NaCl, 2 mM 6-aminohexanoic acid, 1 mM EDTA, 50 mM imidazole-HCl, pH 7) and a proper amount of detergent was added (1.25 μL of dodecylmaltoside (20%) or 3 μL of digitonin (20%)). The mitochondria were solubilized 20 min on ice and debris was removed by 1 h centrifugation at 4 °C and 25,000× *g*. Coomassie dye (5% Coomassie Blue G-250 dye stock solution, suspended 500 mM 6-aminohexanoic acid) was added to the supernatant to give a detergent/dye ratio of 8, and the samples were subjected to electrophoresis in a 4–14% gradient gel. After electrophoresis, either in-gel respiratory complex activities were measured, or Western blot analysis was performed. The measurement of complex I activity the gel was performed according to [19]. Gels were incubated in solution containing 0.14 mM NADH, 1 mg/mL Nitro Blue tetrazolium (NBT) and 0.1 M Tris HCl, pH 7.4, until the appearance of violet bands. The reaction was stopped with 10% acetic acid solution.

### 4.8. CIV Activity

A reaction mixture containing 200 µL buffer (10 mM potassium phosphate buffer, 250 mM sucrose, 1 mg/mL BSA, pH 6.5), 10 µM reduced cytochrome c, and 2 µL dodecylmaltoside solution (20%) was added into a well of 96-well plate. The reaction was started by adding 2–10 × 10^4^ cells, which were frozen/thawed two or three times in isotonic medium before using. To measure the activity of cytochrome c oxidase, the decrease in absorbance at 550 nm was monitored for 3 min using Infinite M200Pro plate reader (Tecan, Männedorf, Switzerland). The specificity of the activity of complex IV was checked by addition of 2 μL of 1M potassium cyanide (KCN). The resulting activity was calculated using formula “CIV activity (nmol cyt c/min/10^6^ cells) = (ΔA(550 nm)/min)/(ε × cell number), where ε = extinction coefficient for reduced cytochrome c 18.5 mM^−1^cm^−1^.

### 4.9. Fractionation of Mitochondrial Ribosomes

Approximately 10^7^ cells were collected, washed with PBS and lysed in 1 mL of 10 mM HEPES pH 7.4, 120 mM KCl, 20 mM magnesium acetate, 5 mM β-mercaptoethanol, 1.7% Triton X-100 for 30 min on ice. After clarification at 25,000× *g* for 30 min, lysates were applied to 10–40% linear sucrose gradient prepared on the same buffer without detergent. After ultracentrifugation at 80,000× *g* for 18 h (SW-40Ti rotor, Beckman Optima XE-90 centrifuge) gradients were fractionated with Gradient Station ip (Biocomp, Fredericton, NB, Canada). Proteins in fractions were sedimentated with methanol/chloroform and applied to Western blotting.

### 4.10. Recombinant PTCD2 Cloning, Expression and Purification

*PTCD2* cDNA was amplified from total HeLa cDNA using 5′-ATCGAGGCCTCTGAGGCCATGGTCCGAGACAGTATGG-3′ and 5′-ATCGAGGCCTGACAGGCCTTAAAGATCTTCTTCGCTAATAAGTTTTTGTTCCTCAGCCAACAGGGACTGG. The fragment was cloned into pET30a vector (Novagen), and verified by Sanger sequencing. Resulting vector was transformed into BL21(DE3)/pLysS *E. coli* strain. 5 mL of LB medium supplemented with 50 ug/mL kanamycin and 20 ug/mL chloramphenicol were used as starting overnight culture grown at 37 °C with agitation; 1L of LB with appropriate antibiotics were inoculated with starting culture and incubated at 30 °C and agitation until OD_600_ reached 0.7–0.9. Expression was induced by adding IPTG up to 0.25 mM, cells were incubated for 4 h at 30 °C. Then cells were collected by centrifugation at 3000× *g* for 10 min, washed with PBS, resuspended in 40 mL of PBS, and lysed by sonication (10 × 15 s at 30% amplitude with 45 s intervals, Branson digital sonifier). Inclusion bodies were pelleted at 25,000× *g* for 20 min and dissolved in 7 mL of 25 mM Tris-HCl pH 7.5, 500 mM NaCl, 25 mM imidazole, 8M urea. After centrifugation (25,000× *g*, 20 min), dissolved inclusion bodies were applied to 1 mL Ni-NTA column (HisTrap, GE Healthcare, Marlborough, MA, USA) connected to AKTA Purifier chromatography system (GE Healthcare, Marlborough, MA, USA) and pre-equilibrated with the same buffer. After extensive washing with the same buffer (40 column volumes), recombinant PTCD2 was eluted with the same buffer supplemented with 300 mM of imidazole. After elution fractions contained PTCD2 were combined and dialyzed against 4 changes of 25 mM sodium phosphate buffer pH 7.5, 150 mM NaCl for 24 h. Protein concentration was measured by Coomassie Protein Assay (Thermo Fisher Scientific, Waltham, MA, USA).

### 4.11. Co-Immunoprecipitation

0.5 mg of HeLa mitochondria were resuspended in solubilizing buffer (50 mM imidazole-HCl (pH 7.0), 50 mM NaCl, 1 mM EDTA, 2 mM 6-aminohexanoic acid) supplemented with 2% dodecylmaltoside. After incubation for 40 min on ice, the insoluble material was removed by centrifugation at 18,000× *g* for 1 h at 4 °C. The supernatant was incubated first with 25 µg of recombinant 6xHis-tagged PTCD2 for 1 h at 4 °C with gentle agitation and then with Ni-NTA-Sepharose (Qiagene, Hilden, Germany). After washing the resin, the proteins were eluted according to the manufacturer’s standard protocol. The eluted proteins were precipitated by methanol/chloroform and subjected to proteomic analysis.

### 4.12. Comparative Proteomics

Proteome analysis was performed as described in [20] with minor modifications. Eluates from the IP were loaded on to the 10% denaturing PAAG and when the bromophenol blue dye passed 5 mm of the separating gel current was stopped and the band containing total concentrated proteins was excised from the gel and sliced into pieces. Excised samples were washed twice with solution 1 (40% acetonitrile and 50 mM NH_4_HCO_3_). The pieces of gel were dried in acetonitrile, rehydrated in solution 2 (100 mM NH_4_HCO_3_ and 20 mM DTT) and incubated for 30 min at 56 °C. Next solution 2 was discarded and the samples were incubated in solution 3 (100 mM NH_4_HCO_3_ and 20 mM iodoacetamide) in the dark for 20 min, washed twice with water, three times with solution 1, dried in acetonitrile and trypsinized with Trypsin Gold (Promega, WI, USA) in 100 mM NH_4_HCO_3_ at 37 °C for 6 h. Peptides were desalted using Pierce C18 Tips, 100 ul according to manufacturer’s instructions. Peptides eluted from the tips were dried on vacuum manifold and solubilized in 0.1% formic acid.

One microgram of peptides in a volume of 1–4 uL was loaded onto the Acclaim μ-Precolumn (0.5 mm × 3 mm, 5 μm particle size, Thermo Scientific) at a flow rate of 10 uL/min for 4 min in an isocratic mode of Mobile Phase C (2% acetonitrile, 0.1% formic acid). Then the peptides were separated with high-performance liquid chromatography (HPLC, Ultimate 3000 Nano LC System, Thermo Scientific, Rockwell, IL, USA) in a 15 cm long C18 column (Acclaim^®^ PepMap™ RSLC inner diameter of 75 μm, Thermo Fisher Scientific, Rockwell, IL, USA). The peptides were eluted with a gradient of buffer B (80% acetonitrile, 0.1% formic acid) at a flow rate of 0.3 μL/min. Total run time was 90 min, which included initial 4 min of column equilibration to buffer A (0.1% formic acid), then gradient from 5 to 35% of buffer B over 65 min, then 6 min to reach 99% of buffer B, flushing 10 min with 99% of buffer B and 5 min re-equilibration to buffer A.

MS analysis was performed at least in triplicate with a Q Exactive HF-X mass spectrometer (Q Exactive HF-X Hybrid Quadrupole-Orbitrap™ Mass spectrometer, Thermo Fisher Scientific, Rockwell, IL, USA). The temperature of capillary was 240 °C and the voltage at the emitter was 2.1 kV. Mass spectra were acquired at a resolution of 120,000 (MS) in a range of 300−1500 *m*/*z*. Tandem mass spectra of fragments were acquired at a resolution of 15,000 (MS/MS) in the range from 100 *m*/*z* to *m*/*z* value determined by a charge state of the precursor, but no >2000 *m*/*z*. The maximum integration time was 50 and 110 ms for precursor and fragment ions, correspondently. AGC target for precursor and fragment ions were set to 1 × 10^6^ and 2 × 10^5^, respectively. An isolation intensity threshold of 50,000 counts was determined for precursor’s selection and up to top 20 precursors were chosen for fragmentation with high-energy collisional dissociation (HCD) at 29 NCE. Precursors with a charge state of +1 and more than +5 were rejected and all measured precursors were dynamically excluded from triggering of a subsequent MS/MS for 20 s.

The obtained raw data were processed using the MaxQuant software [21] with the built-in search engine Andromeda [22]. All samples from one biological replicate were analyzed in one run. Protein sequences of the complete human proteome provided by Uniprot were used for protein identification with Andromeda. Carbamidomethylation of cysteines was set as fixed modification and protein N-terminal acetylation as well as oxidation of methionines was set as variable modification for the peptide search. A maximum mass deviation of 4.5 ppm was allowed for precursor’s identification and 20 ppm were set as match tolerance for fragment identification (acquisition in Orbitrap). Up to two missed cleavages were allowed for trypsin digestion. The false discovery rates (FDR) for peptide and protein identifications were set to 1%. Unique and razor peptides were used for label-free quantification (LFQ).

## Data Availability

Not applicable.
